# Principles and Practice of Medicine

**Published:** 1886-03

**Authors:** 


**Principles and Practice of Medicine.** By the late CHARLES HILTON FAGGE, M. D., F. R. C. P., Physician to, and Lecturer on Pathology at, Guy's Hospital, etc., etc.; including a Section on Cutaneous Diseases, by Pye Smith, M. D., F. R. S.; and Chapters on Cardiac Disease, by Samuel Wilkes, M. D., F. R. S. Vol. I. Philadelphia: P. Blakiston, Son & Co., 1012 Walnut Street. 1886.

Dr. Fagge was not as well known on this side of the Atlantic as some others far less distinguished. As a teacher, scholar and practitioner he had a wide fame; a writer, in the London *Lancet*, says of him: "Fagge was, to my mind, the type of true medical greatness. I believe he was capable of any kind of excellence. His greatness as a physician became evident to observers of character very soon after his brilliant student career had placed him on the staff of Guy's Hospital; he did not merely group already known facts, but he found new facts. Former volumes of Guy's Hospital Reports contain ample and most valuable proof of his greatness as a physician. His power of observation was sustained by immense memory, and brought into action by vivid and constant suggestiveness of intelligence.

He was a physician by grace of nature, and being gifted with a quickness of perception, a genius for clinical facts, and a patience in observation, he was at once recognized as a successful practitioner, and a leading figure in the hospital and among the profession." Those who are fortunate enough to have possession of the first volume of his great work will admit that this is not overpraise, for such a book could have been produced by no ordinary man. The first volume contains 1,040 pages, and the work is to be completed in a second volume of about the same size. We believe the work is destined to take rank as one of the best on "Principles and Practice" in the language. There is a freshness and originality about it, which stamps it as the production of an earnest, laborious, enthusiastic and truthful physician. It is to be deplored that the distinguished author could not have lived to see the full fruition of his labors, but in the hands of its accomplished editor, the work has been prepared for the press, and a few missing chapters supplied in a manner which reflects credit on Dr. Pye Smith. We will look with much interest for the appearance of the second volume.

